# Supplementary material for: Experimental and Computational Methods for Allelic Imbalance Analysis from Single-Nucleus RNA-seq Data
Source: bioRxiv. 2024 Aug 16:2024.08.13.607784. Preprint. [Version 1] doi: 10.1101/2024.08.13.607784 (PMC11343128; doi:10.1101/2024.08.13.607784)

bioRxiv preprint doi: https://doi.org/10.1101/2024.08.13.607784; this version posted August 16, 2024. The copyright holder for this preprint (which was not certified by peer review) is the author/funder, who has granted bioRxiv a license to display the preprint in perpetuity. It is made available under aCC-BY 4.0 International license.

Supplementary Information

Supplementary Figures

Fig. S1 ASE with or without intron-aligned UMIs.

a Comparison of the percentage of UMIs that are phased in each sample either including or excluding introns. **b** Comparison of the estimated allelic imbalance effect size for genes that are significant in either analysis with or without introns for each sample.

Fig. S2 Read length effect on power in each cell type.

Shown for each cell type is the number of genes with significant allelic imbalance for each sample at different read lengths.

Fig. S3. Long-read error rates

For each sequencing technology, shown is the frequency a base disagreed with the reference genome, with either an insertion, deletion, or mismatch. We counted matches (positions were the read matches the reference genome), mismatches (positions where the read maps to the genome but the nucleotide does not match the reference), insertions (locations with an insertion in the read relative to the reference genome), and deletions (locations with a deletion in the read relative to the reference genome, excluding splicing events). We then calculated the number of insertions, deletions, and mismatches divided by the number of matches and plotted the results.

Fig. S4 Isoform-level analysis.

a UpSet plot of the number of peaks assigned to a genomic region according to Sierra's peak annotation. Exon includes 5’ UTR and 3’ UTR peaks. **b** Venn diagram for each sample showing

bioRxiv preprint doi: https://doi.org/10.1101/2024.08.13.607784; this version posted August 16, 2024. The copyright holder for this preprint (which was not certified by peer review) is the author/funder, who has granted bioRxiv a license to display the preprint in perpetuity. It is made available under aCC-BY 4.0 International license.

overlap between genes with significant allelic imbalance and genes with at least one Sierra peak with significant allelic imbalance. **c** Violin plot showing the percentage of phased UMIs coming from the unspliced pre-mRNA transcript for each gene with at least 10 phased UMIs in the isoform level analysis for each sample. **d** Phased UMIs per cell recovered with isoform-level analysis of the MAS-Seq data for both spliced and unspliced reads. **e** Phased UMIs per cell recovered with isoform-level analysis of the short-read data for spliced and unspliced reads with varying lengths of read 2.

Fig. S5 Hybrid selection metrics.

a Percentage of targeted genes with significant allelic imbalance shown for genes with or without a known eQTL. **b** Distance of uniquely mapped reads from hybrid selection baits with and without selection**. c** Comparison of the $\log_2$ of the enrichment score (phased UMIs after selection / phased UMIs before selection) for each selected gene to different metrics for each gene without selection. Total UMI: total UMIs assigned to that gene, Phased UMI: total phased UMIs assigned to that gene, Length: length of the gene in bp, Percentage Phased: percentage of Phased UMI divided by Total UMI, and Percentage Cells: percentage of cells expressing that gene. **d** Violin plots of the enrichment score for genes chosen by each criterion (Methods).

Fig. S6 Additional hybrid selection analysis.

a Boxplots of sequence coverage without selection and with selection. **b** Histogram of sequence coverage with downsampling. Overall, there are an average of 5,365 reads per cell in this dataset. Because the downsampling was based on the number of nuclei reported by Cell Ranger and these results were based on the number of nuclei in the final Seurat object (a smaller number of

bioRxiv preprint doi: https://doi.org/10.1101/2024.08.13.607784; this version posted August 16, 2024. The copyright holder for this preprint (which was not certified by peer review) is the author/funder, who has granted bioRxiv a license to display the preprint in perpetuity. It is made available under aCC-BY 4.0 International license.

nuclei), some samples had more than 5,000 reads per nucleus. Those with fewer than 5,000 reads per nucleus were not downsampled. **c** Comparison of the number of SNP/gene pairs with significant allelic imbalance in each cell type for downsampled selected data and non-downsampled non-selected data. **d** Comparison of effect size estimates for each targeted gene and each cell type in the data with and without selection.

Fig. S7 Sierra peak analysis

Effects of downsampling the number of individuals on Sierra peaks with significant allelic imbalance (**a**) and Sierra peaks with significant allelic imbalance relative to the other peaks in the same genes (**b**). **c** Estimated allelic imbalance for each peak *vs.* estimated allelic imbalance for the associated gene. **d** As in **c**, highlighting *KANSL1* peaks.

Fig. S8 Differential allelic imbalance.

a Runtime of each method for each cell type. Scatter plots comparing the estimated effect size (**b**) and the negative $\log_{10}$ p-values (**c**) from differential allelic imbalance analysis (PD *vs.* healthy controls) for each method in each cell type. All analyses used the PD data with selection and no downsampling.

Fig. S9 Experimental design.

Schematic showing processing of each library with the GTEx samples.

Supplementary Tables

bioRxiv preprint doi: https://doi.org/10.1101/2024.08.13.607784; this version posted August 16, 2024. The copyright holder for this preprint (which was not certified by peer review) is the author/funder, who has granted bioRxiv a license to display the preprint in perpetuity. It is made available under aCC-BY 4.0 International license.

Supplementary Table 1 QC metrics reported by STARSolo and the ASE pipeline for GTEx samples

Supplementary Table 2 Peak- and gene-level allelic imbalance results with GTEx data

Supplementary Table 3 List of GTEx samples for long- and short-read data

Supplementary Table 4 Allelic imbalance results for PD data with and without selection

Supplementary Table 5 Selected genes in PD and GTEx data with gene-level QC metrics

Supplementary Table 6 Targeted regions in selection experiments for PD and GTEx data

Supplementary Table 7 Annotated Sierra peaks for PD and GTEx data

Supplementary Table 8 Key Resource Table

bioRxiv preprint doi: https://doi.org/10.1101/2024.08.13.607784; this version posted August 16, 2024. The copyright holder for this preprint (which was not certified by peer review) is the author/funder, who has granted bioRxiv a license to display the preprint in perpetuity. It is made available under aCC-BY 4.0 International license.

Supp. Fig. 1

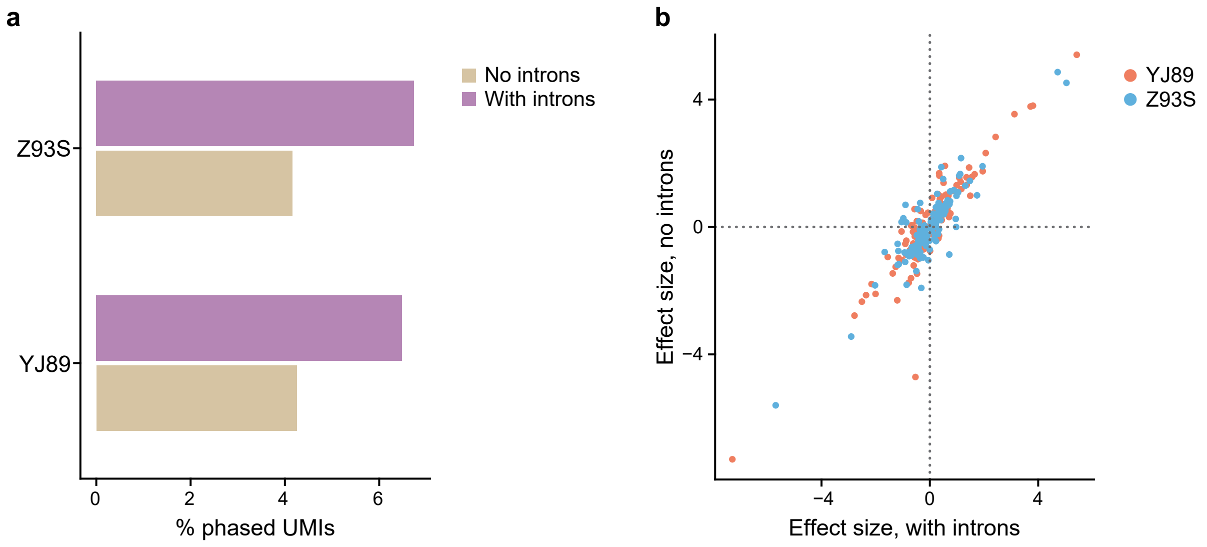

bioRxiv preprint doi: https://doi.org/10.1101/2024.08.13.607784; this version posted August 16, 2024. The copyright holder for this preprint (which was not certified by peer review) is the author/funder, who has granted bioRxiv a license to display the preprint in perpetuity. It is made available under aCC-BY 4.0 International license.

Supp. Fig. 2

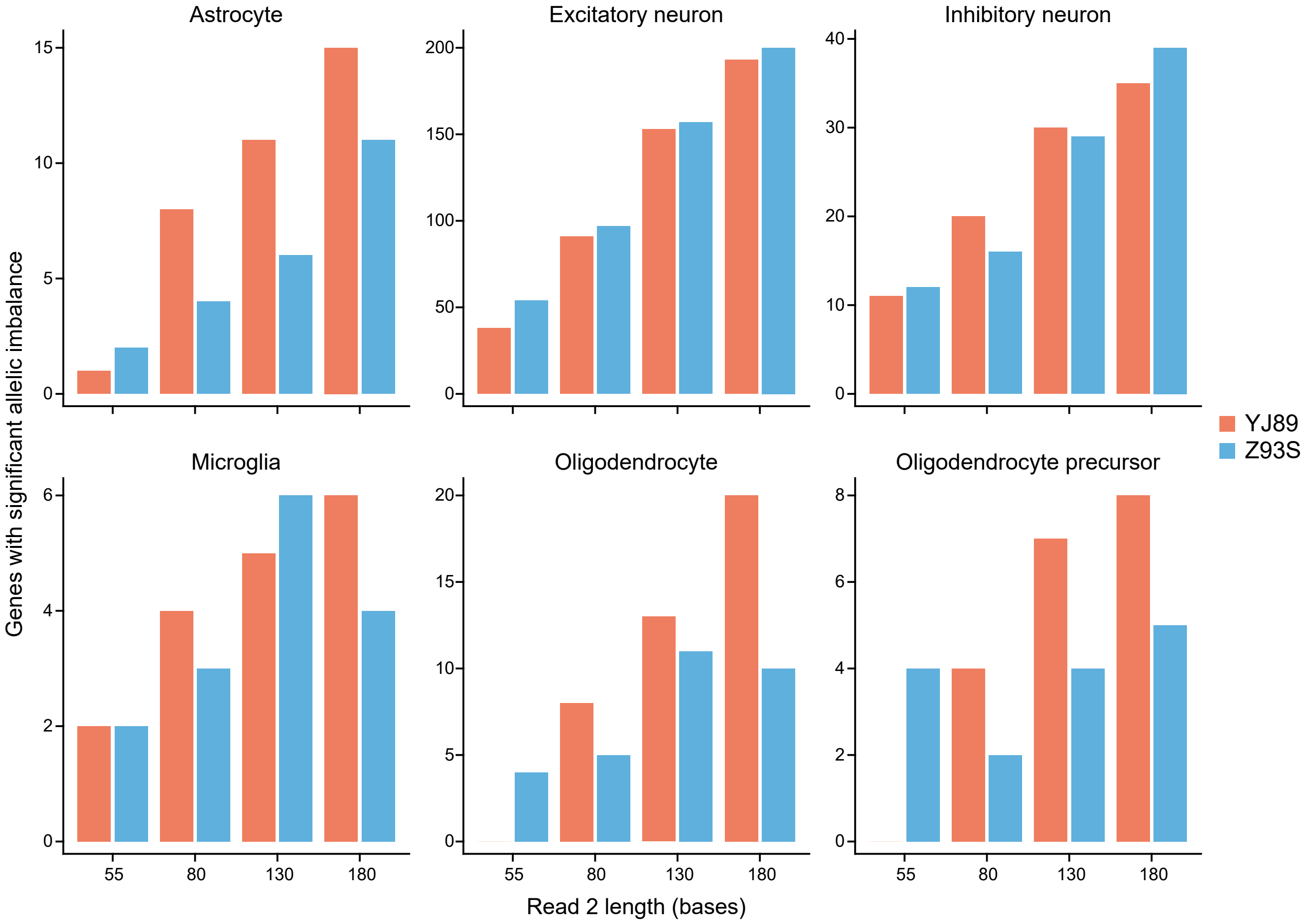

bioRxiv preprint doi: https://doi.org/10.1101/2024.08.13.607784; this version posted August 16, 2024. The copyright holder for this preprint (which was not certified by peer review) is the author/funder, who has granted bioRxiv a license to display the preprint in perpetuity. It is made available under aCC-BY 4.0 International license.

Supp. Fig. 3

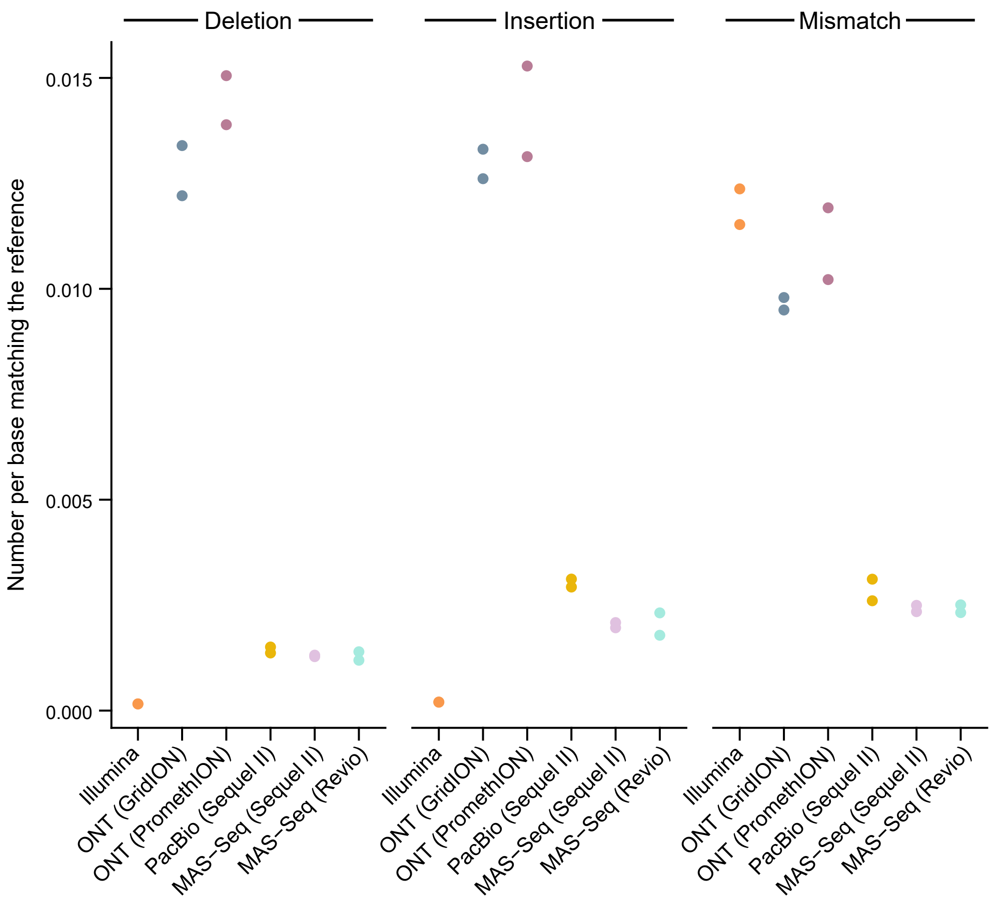

bioRxiv preprint doi: https://doi.org/10.1101/2024.08.13.607784; this version posted August 16, 2024. The copyright holder for this preprint (which was not certified by peer review) is the author/funder, who has granted bioRxiv a license to display the preprint in perpetuity. It is made available under aCC-BY 4.0 International license.

Supp. Fig. 4

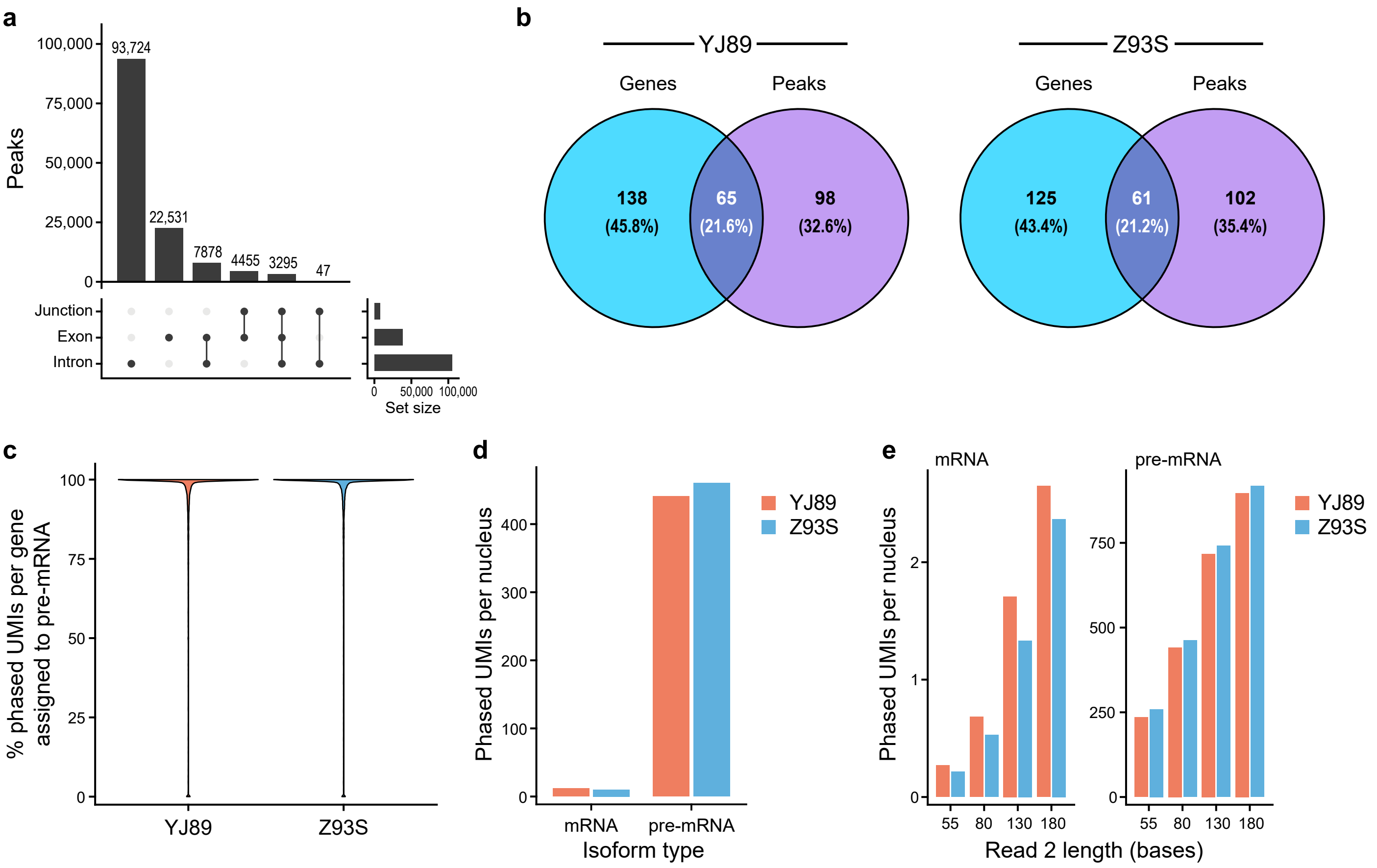

bioRxiv preprint doi: https://doi.org/10.1101/2024.08.13.607784; this version posted August 16, 2024. The copyright holder for this preprint (which was not certified by peer review) is the author/funder, who has granted bioRxiv a license to display the preprint in perpetuity. It is made available under aCC-BY 4.0 International license.

Supp. Fig. 5

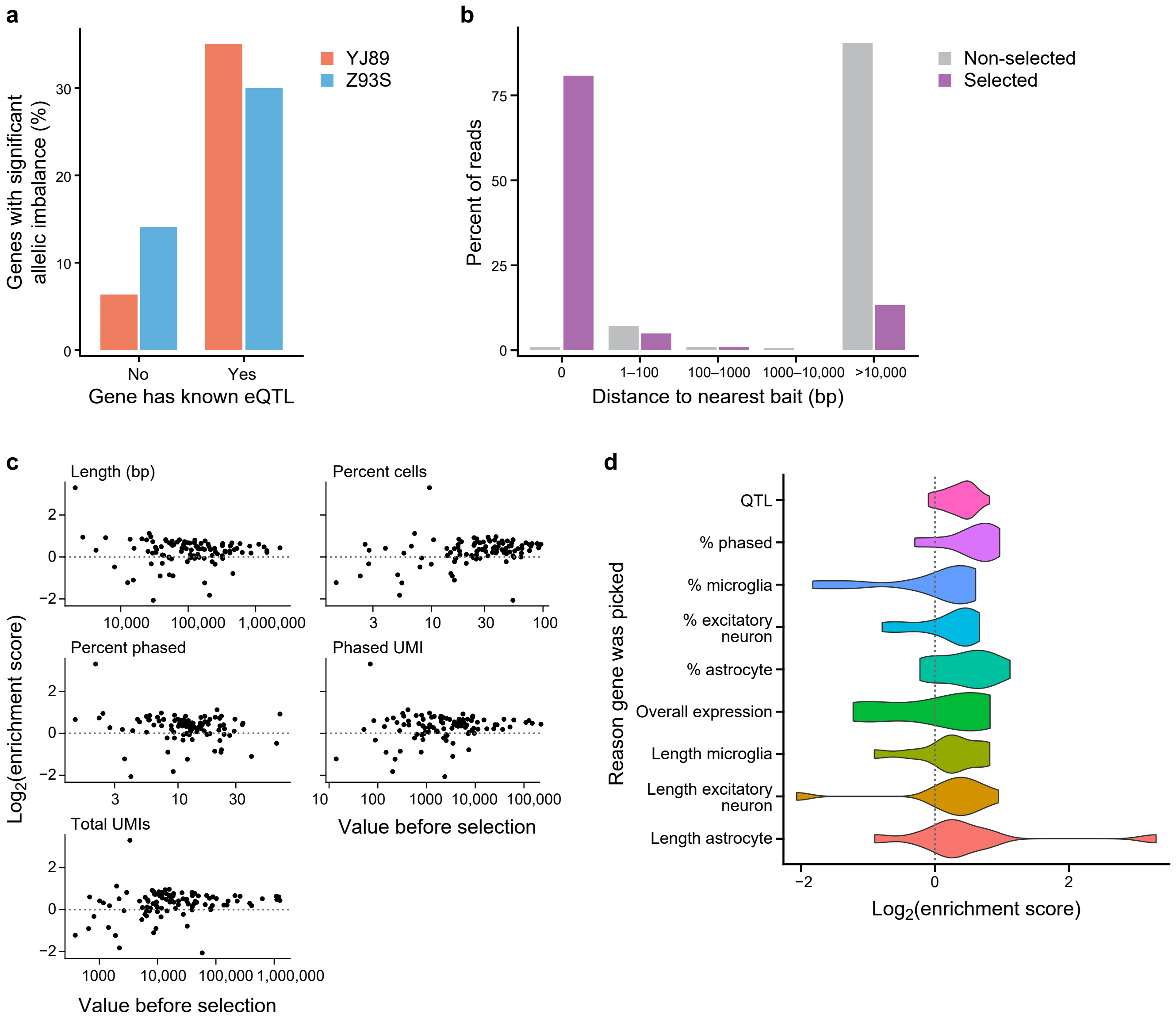

bioRxiv preprint doi: https://doi.org/10.1101/2024.08.13.607784; this version posted August 16, 2024. The copyright holder for this preprint (which was not certified by peer review) is the author/funder, who has granted bioRxiv a license to display the preprint in perpetuity. It is made available under aCC-BY 4.0 International license.

Supp. Fig. 6

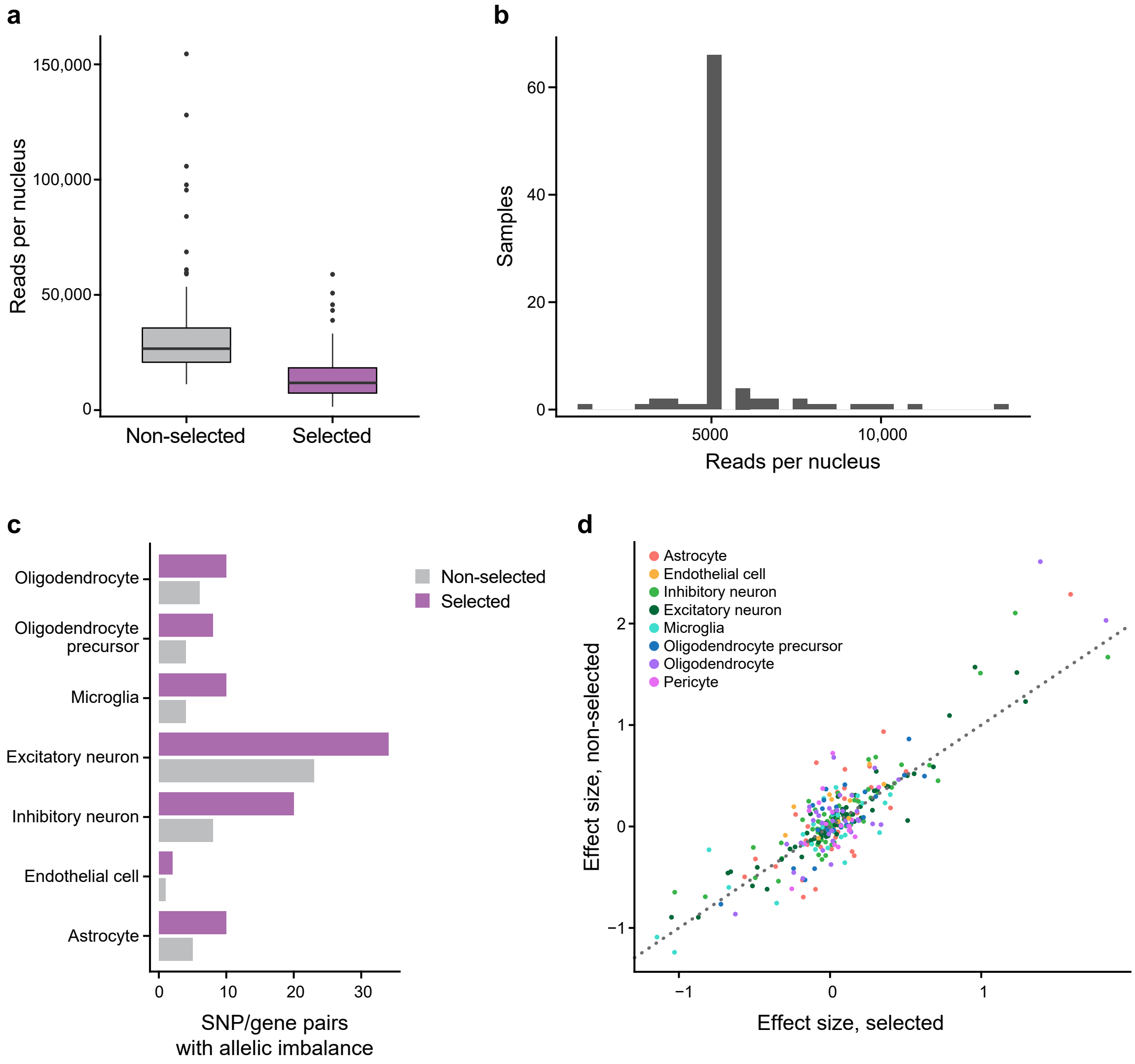

bioRxiv preprint doi: https://doi.org/10.1101/2024.08.13.607784; this version posted August 16, 2024. The copyright holder for this preprint (which was not certified by peer review) is the author/funder, who has granted bioRxiv a license to display the preprint in perpetuity. It is made available under aCC-BY 4.0 International license.

Supp. Fig. 7

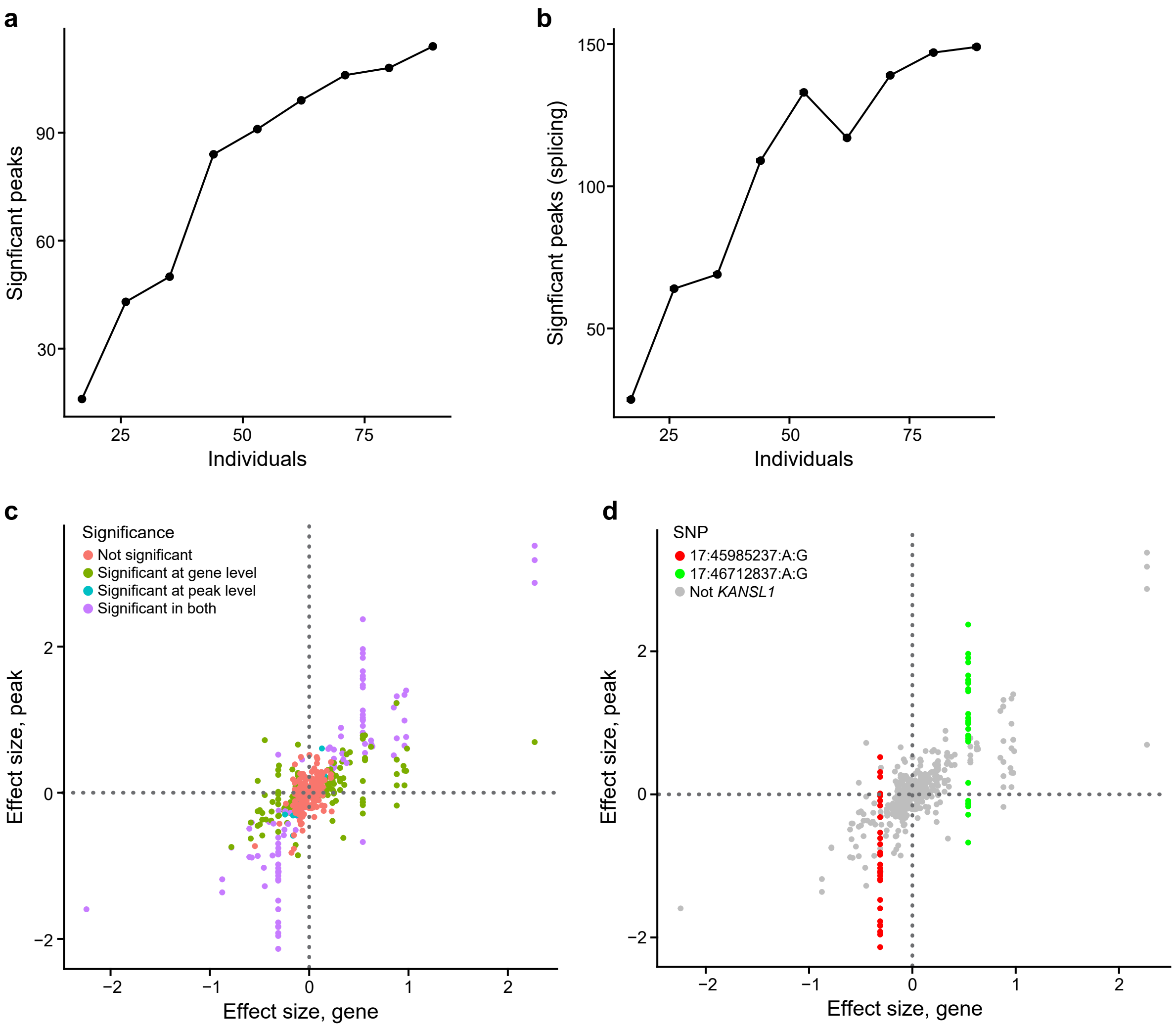

bioRxiv preprint doi: https://doi.org/10.1101/2024.08.13.607784; this version posted August 16, 2024. The copyright holder for this preprint (which was not certified by peer review) is the author/funder, who has granted bioRxiv a license to display the preprint in perpetuity. It is made available under aCC-BY 4.0 International license.

Supp. Fig. 8

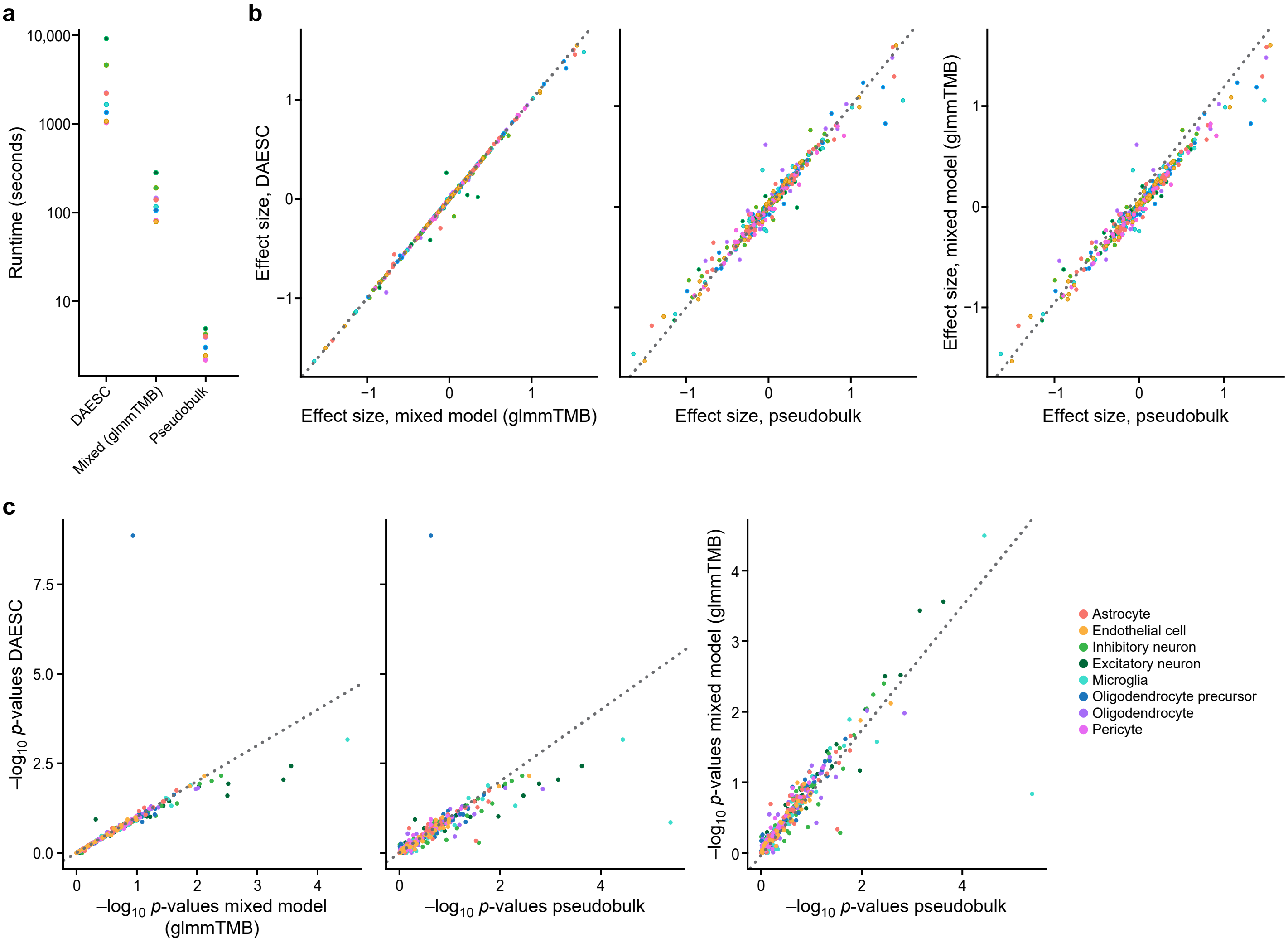

bioRxiv preprint doi: https://doi.org/10.1101/2024.08.13.607784; this version posted August 16, 2024. The copyright holder for this preprint (which was not certified by peer review) is the author/funder, who has granted bioRxiv a license to display the preprint in perpetuity. It is made available under aCC-BY 4.0 International license.

Supp. Fig. 9

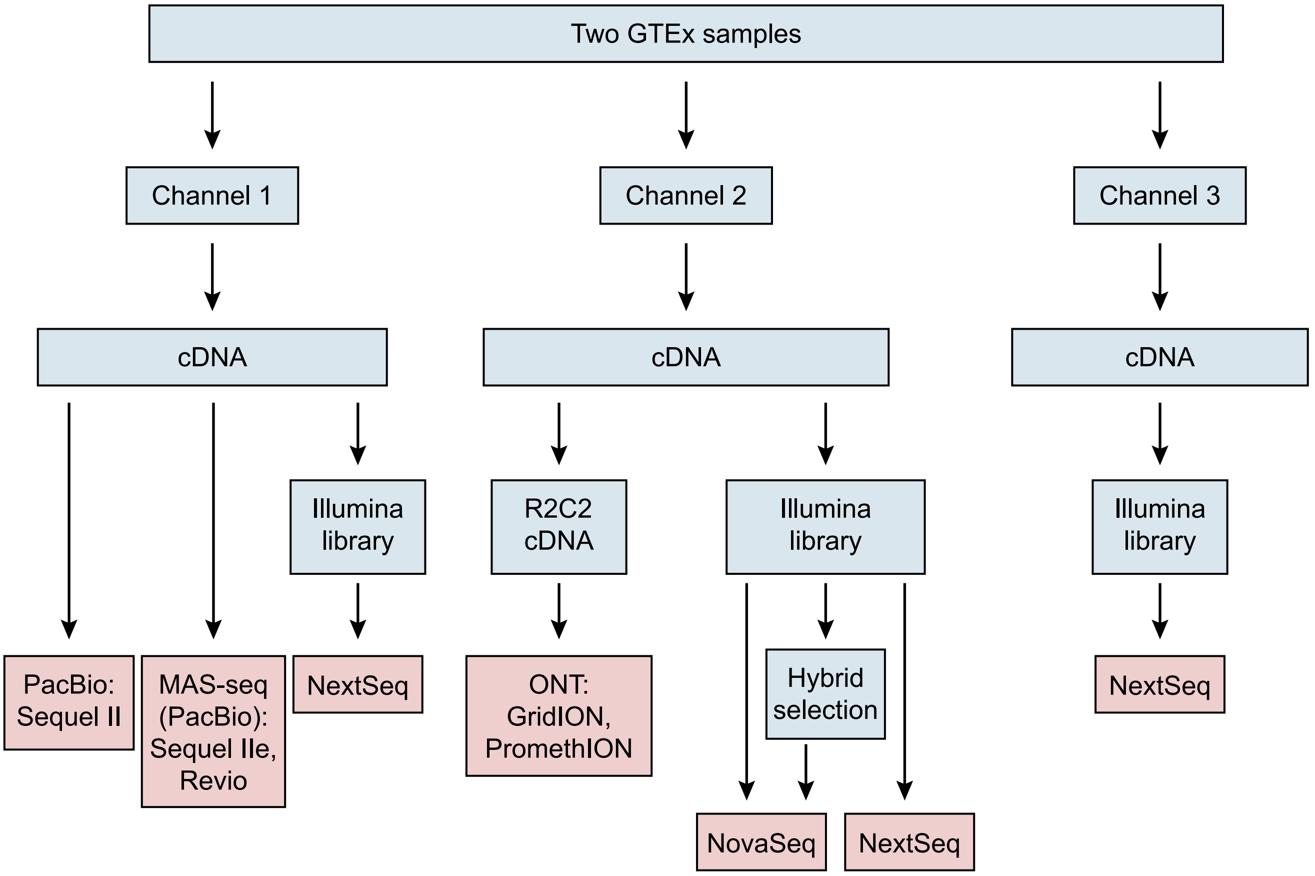

Supplement: Supplementary file 1 [file NIHPP2024.08.13.607784v1-supplement-9.pdf]
